# A Rapid and Highly Predictive *in vitro* Screening Platform for Osteogenic Natural Compounds Using Human Runx2 Transcriptional Activity in Mesenchymal Stem Cells

**DOI:** 10.3389/fcell.2020.607383

**Published:** 2021-01-08

**Authors:** Li-Tzu Wang, Yu-Wei Lee, Chyi-Huey Bai, Hui-Chun Chiang, Hsiu-Huan Wang, B. Linju Yen, Men-Luh Yen

**Affiliations:** ^1^Department of Obstetrics and Gynecology, National Taiwan University (NTU) Hospital and College of Medicine, Taipei, Taiwan; ^2^Regenerative Medicine Research Group, Institute of Cellular and System Medicine, National Health Research Institutes, Zhunan, Taiwan; ^3^School of Public Health, College of Public Health, Taipei Medical University, Taipei, Taiwan; ^4^Department of Public Health, School of Medicine, College of Medicine, Taipei Medical University, Taipei, Taiwan; ^5^Department of Obstetrics and Gynecology, Cathay General Hospital Shiji, New Taipei City, Taiwan

**Keywords:** osteoporosis, Runx2, luciferase promoter assay, mesenchymal stem cells, drug screening platform

## Abstract

The rapid aging of worldwide populations had led to epidemic increases in the incidence of osteoporosis (OP), but while treatments are available, high cost, adverse effects, and poor compliance continue to be significant problems. Naturally occurring plant-based compounds including phytoestrogens can be good and safe candidates to treat OP, but screening for osteogenic capacity has been difficult to achieve, largely due to the requirement of using primary osteoblasts or mesenchymal stem cells (MSCs), the progenitors of osteoblasts, to conduct time-consuming *in vitro* and *in vivo* osteogenic assay. Taking advantage of MSC osteogenic capacity and utilizing a promoter reporter assay for Runx2, the master osteogenesis transcription factor, we developed a rapid *in vitro* screening platform to screen osteogenic small molecules including natural plant-based compounds. We screened eight plant-derived compounds from different families including flavonoids, polyphenolic compounds, alkaloids, and isothiocyanates for osteogenic capacity using the human RUNX2-promoter luciferase reporter (hRUNX2-luc) transduced into the mouse MSC line, C3H10T1/2, with daidzein—a well-studied osteogenic phytoestrogen—as a positive control. Classical *in vitro* and *in vivo* osteogenesis assays were performed using primary murine and human bone marrow MSCs (BMMSCs) to validate the accuracy of this rapid screening platform. Using the MSC/hRUNX2-luc screening platform, we were able not only to shorten the selection process for osteogenic compounds from 3∼4 weeks to just a few days but also simultaneously perform comparisons between multiple compounds to assess relative osteogenic potency. Predictive analyses revealed nearly absolute correlation of the MSC/hRUNX2-luc reporter platform to the *in vitro* classical functional assay of mineralization using murine BMMSCs. Validation using human BMMSCs with *in vitro* mineralization and *in vivo* osteogenesis assays also demonstrated nearly absolute correlation to the MSC/hRUNX2-luc reporter results. Our findings therefore demonstrate that the MSC/hRUNX2 reporter platform can accurately, rapidly, and robustly screen for candidate osteogenic compounds and thus be relevant for therapeutic application in OP.

## Introduction

With the aging and increased life expectancy of worldwide populations, the incidence of osteoporosis (OP) in both men and postmenopausal women is reaching epidemic proportions. A progressive systemic disease in which bone mineral density is decreased, OP significantly increases fracture risk, with risks of OP-related fracture in women and men over 50 years old estimated to be 30 and 20%, respectively (Siris et al., 2001; Cooper et al., 2011). Among the most serious OP-related fracture are vertebral and hip fractures, which have devastating economic as well as health consequences with mortality as high as one in three patients within 1 year after a hip fracture in some estimates (Burge et al., 2007; Katsoulis et al., 2017). Women in particular are at risk for OP decades before men, due to the loss of estrogen at menopause (Rosen, 2005). The simplest therapy for postmenopausal OP therefore has been to replace the lost endogenous sex hormone with exogenous estrogen or hormone replacement therapy (HRT). However, long-term use of HRT is now known to be associated with increasing breast cancer and vascular disease risk (Hulley et al., 1998; Rossouw et al., 2002), necessitating other therapeutic options. The most common treatments act on bone-resorbing osteoclasts to mitigate bone loss, such as bisphosphonates and denosumab. While effective, such agents are slow to act, taking several months to years to see significant effects. Bone anabolic agents including selective estrogen receptor modulators (SERMs) and teriparatide, a form of parathyroid hormone, target bone-producing osteoblasts and induce bone growth more rapidly, but all OP treatments are difficult to adhere to due to numerous side effects which range from discomfort to cancer risk to—ironically—atypical fracture risk (Black and Rosen, 2016). In addition, many of the newer therapies are prohibitively expensive, bringing into question widespread use especially in developing nations where OP is increasing rapidly (Handa et al., 2008; Mailankody and Prasad, 2014). Given the global reach of OP, more affordable therapeutic options are clearly necessary.

Naturally occurring plant-based compounds have been known to harbor therapeutic properties including osteogenesis. Phytoestrogens, which are naturally occurring non-steroidal plant compounds, are especially good candidates given their ability to exert beneficial agonistic effects on alleviating menopausal symptoms and bone loss (Dutertre and Smith, 2000; Lv et al., 2018; Geng et al., 2019; Zhou et al., 2020), without increasing estrogenic cancer risks (Adlercreutz, 2002; Horn-Ross et al., 2003; Anderson et al., 2013; Yang et al., 2019). Reports on the osteogenic agonism of individual phytoestrogens have been varied, ranging from strong agonism to antagonism with induction of adipogenesis even for the same compound despite the structural similarity to estrogen (Dang and Lowik, 2005). Moreover, classical osteogenesis assays are time-consuming, with both *in vitro* and *in vivo* functional assays requiring several weeks of time to complete. In addition, all these assays are performed with primary cells whether it be osteoblasts, pre-osteoblasts, or mesenchymal stem cells (MSCs) which are the progenitors of osteogenic cells (Zheng et al., 2018, 2019; Abdelrazik et al., 2019; Lavrentieva et al., 2020), all which require primary isolation and can lose osteogenic capacity when senescent after prolonged *in vitro* passaging (Moerman et al., 2004; Ho et al., 2013; Liu et al., 2020). Thus, development of a robust and rapid screening platform for selection of phytoestrogens and other plant-based compounds with strong osteogenic agonistic properties is sorely needed.

Runx2 is the master transcription factor controlling osteogenesis (Ducy et al., 1997; Komori et al., 1997), and strong osteogenic agonists including estrogens, Wnt/β-catenin, bone morphogenic proteins (BMPs), and sirtuins consistently upregulate the activity of this critical gene (Lee et al., 2000; McCarthy et al., 2003; Hill et al., 2005; Zainabadi et al., 2017). We therefore developed a rapid and robust *in vitro* osteogenesis MSC-based screening platform using a luciferase reporter of the human RUNX2 promoter (hRUNX2-luc) transduced into an immortalized but non-cancerous murine MSC line. Classical *in vitro* and *in vivo* osteogenesis assays using primary murine and human MSCs were performed to validate the findings of this *in vitro* platform. High correlation of the cell-based hRUNX2-luc *in vitro* screening system was found with both *in vitro* and *in vivo* mineralization assays, demonstrating this method to be a feasible and robust platform for rapid selection of phytoestrogens and osteogenic natural compounds.

## Materials and Methods

### Cell Culture and Differentiation Studies

Mouse C3H10T1/2 (C3H) mesenchymal progenitor/stem cells (Taylor and Jones, 1979) were obtained from American Type Culture Collection (ATCC, Manassas, VA, United States) and maintained in Basal Medium Eagle medium (BMEM) (Invitrogen-Thermo Fisher Scientific, MA, United States) with 10% FBS (Hyclone-Thermo Fisher Scientific), 100 U/ml of penicillin/streptomycin, 2 mM L-glutamine (all from Gibco-Thermo Fisher Scientific). Murine bone marrow (BM)-derived MSCs were isolated from C57BL/6J strain (National Laboratory Animal Center, Taipei, Taiwan), and human BMMSCs were obtained from commercial sources (Promocell, Heidelberg, Germany) and cultured in complete medium (CM) consisting of low-glucose Dulbecco’s Modified Eagle’s medium (DMEM) (Invitrogen-Thermo Fisher Scientific) with 10% FBS, 100 U/ml of penicillin/streptomycin, and 2 mM L-glutamine. MSC mesodermal differentiation assays were performed as previously reported (Tseng et al., 2011; Yen et al., 2011). Adipogenic differentiation medium (AM) consisted of in CM with 0.5 mM isobutyl-methylxanthine, 1 μM dexamethasone, and 0.1 μM insulin (all obtained from Sigma-Aldrich, St Louis, MO, United States), whereas osteogenic differentiation medium (OM) consisted of CM with 0.2 mM ascorbate, 10 nM dexamethasone, and 10 mM β-glycerophosphate (all from Sigma-Aldrich). For promoter assays, cells were cultured in either CM, AM, or OM for 2 days after transfection. For functional osteogenic differentiation and assays, cells were cultured in OM for 3 weeks and replaced with fresh medium every 3 days. Isolated natural-occurring compounds were dissolved in various vehicles (which were also used as controls) as recommended by the manufacturer in the following manner: daidzein (purity ≥ 95%) and apigenin (purity ≥ 98%) were dissolved in dimethyl sulfoxide (DMSO), while baicalein (purity ≥ 95%), caffeic acid phenylethyl ester (CAPE, purity ≥ 98%), capsaicin (purity ≥ 95%), curcumin (purity ≥ 90%), epicatechin (purity ≥ 90%), naringenin (purity ≥ 98%), and sulforaphane (purity ≥ 98%) were dissolved in ethanol. Doses of each compound were added as indicated, and all compounds were purchased from Cayman Chemical (Ann Arbor, MI, United States).

### Cytotoxicity Assay

All compounds were tested at various doses for their cytotoxic effect on C3H cells by colorimetric analyses of cell viability with the 3-(4,5-dimethylthiazol-2-yl)-2,5-diphenyltetrazolium bromide (MTT) assay (GoldBio, St Louis, MO, United States) according to the manufacturer’s recommendations.

### Promoter Luciferase Reporter Assay

The hRUNX2-luc reporter plasmid was constructed as we previously reported (Tseng et al., 2011). Briefly, the upstream of human RUNX2 promoter (−1,557/ + 32) was constructed into pGL3-basic Luc (Promega, San Luis Obispo, CA, United States). Murine MSC line C3H cells (4 × 10^4^/well) were maintained in 24-well plates and then co-transfected with a 1 μg plasmid mixture of hRUNX2-luc and CMV-driven β-galactosidase construct plasmid (pCMV-β-gal) at a 9:1 ratio using DNAFect LT transfection reagent (ATGCell, Edmonton, Alberta, Canada) according to the manufacturer’s recommendation. After 24 h, the media were replaced with fresh CM, osteogenic medium (OM) or adipogenic medium (AM) and tested compounds for another 48 h. Cell extractions were prepared, and luciferase activity was measured in a microplate luminometer using the Promega Luciferase Assay System (Promega, Madison, WI, United States) standardized against β-galactosidase activity.

### Alkaline Phosphatase Activity

Alkaline phosphatase (ALP) activity assay was performed as previously reported (Tseng et al., 2011). Briefly, cells were lysed by protein lysis buffer without protease inhibitor, and cellular ALP activity was assessed by incubating the protein lysates with substrate p-nitrophenylphosphate (pNPP, Sigma-Aldrich) at 37°C for 30 min, with the colorimetrical reaction measured by absorbance at 405 nm and normalized to corresponding protein amounts.

### Alizarin Red Staining

Alizarin Red (AR) staining and quantification was performed to analyze calcium deposition as previously described (Tseng et al., 2011). Briefly, cells were fixed with 100% methanol for 30 min, washed with boric acid buffer (0.1 M, pH 4.0), and stained with AR solution (40 mM, pH 4.2, Sigma-Aldrich) for 30 min. Unbound AR stain was washed with boric acid buffer twice, and then distilled water until calcium deposits were visualized. To quantify mineralization, elution of AR stain with 10% cetylpyridinium chloride (Sigma-Aldrich) was performed and quantified using spectrophotometric analysis by reading absorbance at 520 nm.

### Quantitative PCR

Quantitative PCR (qPCR) was performed as previously reported (Ho et al., 2013). RNA was extracted with TRIzol reagent (Invitrogen-Thermo Fisher Scientific), and cDNA synthesis was performed with ReverTra Ace set (TOYOBO, Osaka, Japan) according to the manufacturer’s protocols. qPCR was carried out using the SYBR Green Supermix Taq Kit (Bio-Rad Laboratories, Hercules, CA, United States) and performed on the ABI Real-time PCR 7500 System according to the manufacturer’s instructions (Applied Biosystems Inc., Carlsbad, CA, United States). Primers for amplifying human osteogenic and adipogenic genes are as follows: GAPDH (internal control), forward primer 5′-GTGGACCTGACCTGCCGTCT-3′, reverse primer 5′-GGAGGAGTGGGTGTCGCTGT-3′; RUNX2, forward primer 5′-CCAGATGGGACTGTGGTTACTG-3′, reverse primer 5′-TTCCGGAGCTCAGCAGAATAA-3′; C/EBPβ, forward primer 5′-AAACTCTCTGCTTCTCCCTCTG-3′, reverse primer 5′-GTTGCGTCAGTCCCGTGTA-3′.

### *In vivo* Ectopic Bone Formation Assay

Animal experimentation was performed using protocols approved by the Institutional Animal Care and Use Committee. Human BMMSCs (2 × 10^6^ cells) were treated with various compounds for 2 days then mixed with 300 μl Matrigel matrix (BD Biosciences, San Jose, CA, United States) for subcutaneous injection into the dorsal surface of 6-week-old NOD/SCID mice as previously reported (Reinisch et al., 2015). After 5 weeks, the implants were harvested and fixed with 10% formalin overnight. Then, paraffin embedding was performed and 5-μm sections were prepared for histological analyses with hematoxylin and eosin (H&E) staining.

### Statistical Analysis

For comparisons between two groups, Student’s *t*-test was used for analyses, and for comparisons between multiple groups, ANOVA was used for analyses. Data was presented as mean ± SD. Goodness of fit (with Chi square value) was used to assess the predictive ability of the MSC/hRUNX2-luc screening results to the data of the functional assays. *p* < 0.05 was defined as statistically significant. Analyses were performed using GraphPad Prism software (San Diego, CA, United States).

## Results

### Development of an MSC-Based Rapid Screening Platform Using hRUNX2 Promoter Activity for Selection of Osteogenic Natural Compounds

**FIGURE 1:**
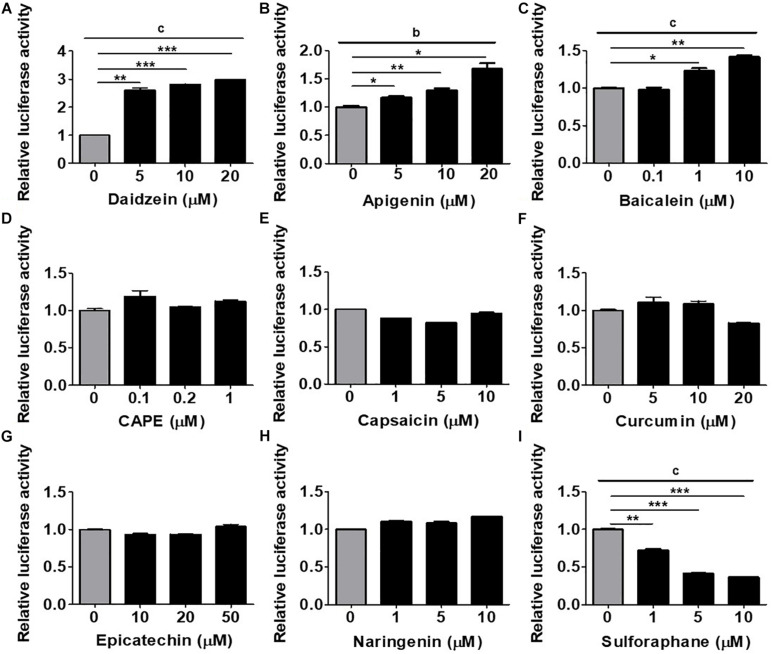
Assessment of an MSC-based rapid screening platform using the human RUNX2 (hRUNX2) proximal promoter activity for selection of osteogenic natural compounds. **(A)** hRUNX2-promoter activity in murine C3H10T1/2 (C3H) mesenchymal stem cell (MSC) line cultured under osteogenic conditions as assessed by measurement of luminometric luciferase signals 48 h after treatment with or without **(A)** daidzein (5–20 μM), **(B)** apigenin (5–20 μM), **(C)** baicalein (0.1–10 μM), **(D)** CAPE (0.1–1 μM), **(E)** capsaicin (1–10 μM), **(F)** curcumin (5–20 μM), **(G)** epicatechin (10–50 μM), **(H)** naringenin (1–10 μM), or **(I)** sulforaphane (1–10 μM). All data were normalized to the luminometric luciferase signal in osteogenic medium (OM)-culturing C3H MSCs without compound treatment (*n* = 3 for each group). Data are expressed as mean ± SD. *, *p* < 0.05; **, *p* < 0.01; and ***, *p* < 0.001 as analyzed by Student’s *t-*test; a, *p* < 0.05; b, *p* < 0.01; and c, *p* < 0.001 as analyzed by ANOVA.

To establish an *in vitro* platform for screening osteogenic compounds, we utilized the hRUNX2-luc reporter vector which was transfected into a well-documented MSC line, C3H MSCs (Taylor and Jones, 1979). To demonstrate that hRUNX2 promoter activity is strongly elicited during osteogenesis, we cultured hRUNX2-luc-transfected C3H cells in CM, AM, or OM conditions. We found that not only is hRUNX2 transcriptional activity significantly activated in OM compared to CM conditions but that the activity is significantly decreased in AM compared to CM conditions (Supplementary Figure 1). To determine the optimal doses of the tested compounds, we performed dose-dependent cytotoxicity assays with all compounds at various concentrations on C3H cells cultured in CM and OM (Supplementary Figure 2). The phytoestrogen daidzein has been well documented in *in vitro* studies to have strong osteogenic inductive properties via interactions with estrogen receptors given its structural similarities to estrogen (Dang and Lowik, 2004; Schilling et al., 2014). We confirmed these previous results, finding that daidzein at the concentrations of 5, 10, and 20 μM significantly induced hRUNX2-luc activity in C3H cells cultured under OM conditions for 48 h (Figure 1A), and therefore used this estrogenic isoflavone as a positive osteogenic compound in subsequent assays. We then used this MSC-based platform to screen the osteogenic efficacy of a wide variety of plant-based compounds with reports of medicinal efficacy: the flavones apigenin and baicalein; the polyphenolic compounds caffeic acid phenethy ester (CAPE) and curcumin; the flavonols epicatechin and naringenin; capsaicin, an alkaloid; and sulforaphane, an isothiocyanate. We found that both apigenin (Figure 1B) and baicalein (Figure 1C) significantly increased hRUNX2-luc activity in C3H cells in a dose-dependent fashion. On the other hand, CAPE (Figure 1D), capsaicin (Figure 1E), curcumin (Figure 1F), epicatechin (Figure 1G), and naringenin (Figure 1H) did not activate hRUNX2-luc. Sulforaphane unexpectedly decreased hRUNX2-luc activity significantly in a dose-dependent manner (Figure 1I). These findings demonstrate that hRUNX2 transcriptional activity in MSCs was rapidly and strongly upregulated by daidzein, a known potent osteogenic inducer, and was also significantly upregulated by apigenin and baicalein while decreased by sulforaphane.

### Validation of hRUNX2-Promoter Activation With *in vitro* ALP Activity, an Early-Stage Assay for Osteogenesis

**FIGURE 2:**
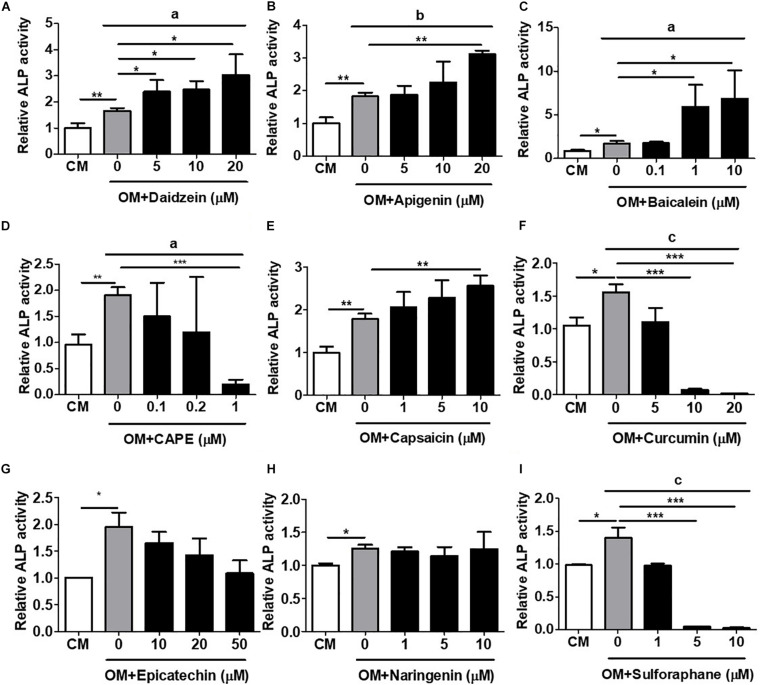
Validation of hRUNX2-promoter activation with *in vitro* alkaline phosphatase (ALP) activity, an early-stage assay for osteogenesis. ALP activity was assessed in primary murine bone marrow (BM) MSCs at 8 days cultured in complete medium (CM) or OM with or without treatment of **(A)** daidzein (5–20 μM), **(B)** apigenin (5–20 μM), **(C)** baicalein (0.1–10 μM), **(D)** CAPE (0.1–1 μM), **(E)** capsaicin (1–10 μM), **(F)** curcumin (5–20 μM), **(G)** epicatechin (10–50 μM), **(H)** naringenin (1–10 μM), or **(I)** sulforaphane (1–10 μM). All data were normalized to the enzymatic activity of cellular ALP in CM-cultured BMMSCs (*n* = 3 for each group). Data are expressed as mean ± SD. *, *p* < 0.05; **, *p* < 0.01; and ***, *p* < 0.001 as analyzed by Student’s *t-*test; a, *p* < 0.05; b, *p* < 0.01; and c, *p* < 0.001 as analyzed by ANOVA.

To validate this rapid and MSC-based osteogenic compound screening platform, we performed a number of classical *in vitro* and *in vivo* osteogenesis functional assays using primary isolated murine and human BMMSCs. We first assessed the induction of *in vitro* cellular ALP activity, an early osteogenic biomarker, with primary murine BMMSCs. Using daidzein again as a positive control, we found that addition of this phytoestrogen to OM-cultured BMMSCs after 1 week significantly increased ALP activity in a dose-dependent fashion over that of BMMSCs cultured in OM only (Figure 2A). Addition of apigenin at the dose of 20 μM (Figure 2B) or baicalein at doses of 1 and 10 μM (Figure 2C) to OM also significantly increased ALP activity in BMMSCs over OM-only culture; these findings are indicative of osteogenic differentiation and consistent to the activation of hRUNX2 promoter activity by these two compounds (Figures 1B,C). Capsaicin was the only other compound which significantly increased BMMSC ALP activity (Figure 2E), which is in contrast to its lack of hRUNX2 promoter activation (Figure 1E). All other tested compounds either decreased ALP activity in OM-cultured BMMSCs, including CAPE (Figure 2D), curcumin (Figure 2F), epicatechin (Figure 2G), and sulforaphane (Figure 2I), or had no effect like naringenin (Figure 2H); these five compounds also did not activate hRUNX2 promoter activity. Collectively, these results show that daidzein, apigenin, and baicalein increase both ALP activity and hRUNX2 transcriptional activity, while sulforaphane decrease activities of both osteogenic assays, while the other five compounds demonstrate either no or inconsistent effects in both assays.

### Validation of hRUNX2-Promoter Activation With *in vitro* Mineralization, a Late-Stage Assay for Osteogenesis

**FIGURE 3:**
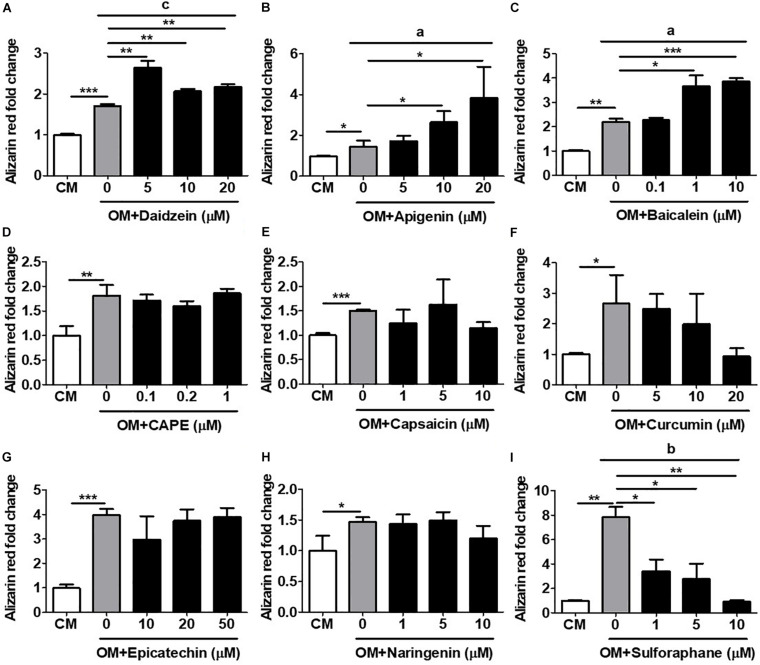
Validation of hRUNX2-promoter activation with *in vitro* mineralization, a late-stage assay for osteogenesis. *In vitro* calcium deposition and mineralization in mouse BMMSCs was assessed by Alizarin Red (AR) staining with elution for quantification after 3 weeks of culture in CM or OM with addition of **(A)** daidzein (5–20 μM), **(B)** apigenin (5–20 μM), **(C)** baicalein (0.1–10 μM), **(D)** CAPE (0.1–1 μM), **(E)** capsaicin (1–10 μM), **(F)** curcumin (5–20 μM), **(G)** epicatechin (10–50 μM), **(H)** naringenin (1–10 μM), or **(I)** sulforaphane (1–10 μM). All data were normalized to AR levels in CM-cultured BMMSCs (*n* = 3 for each group). Data are expressed as mean ± SD. *, *p* < 0.05; **, *p* < 0.01; and ***, *p* < 0.001 as analyzed by Student’s *t-*test; a, *p* < 0.05; b, *p* < 0.01; and c, *p* < 0.001 as analyzed by ANOVA.

The most definitive *in vitro* functional assay for osteogenesis is calcium deposition and mineralization, a late-stage event in osteogenesis which typically requires several weeks of time to perform (Gregory et al., 2004). We therefore assess the capacity of all compounds to induce *in vitro* mineralization in primary isolated murine BMMSCs undergoing osteogenesis using AR staining with subsequent quantification. Using daidzein as a positive control, we found that addition of this compound at any dose to OM-cultured BMMSCs after 3 weeks’ time led to significantly increased calcium deposition, compared to BMMSCs cultured in OM only (Figure 3A). Addition of either apigenin at 10 and 20 μM (Figure 3B) or baicalein at 1 and 10 μM (Figure 3C) to OM-cultured BMMSCs also significantly increased the levels of AR staining compared to BMMSCs cultured in OM only, with both compounds eliciting a more robust mineralization response and in a dose-dependent fashion than daidzein. These results are in line with the ALP activity and hRUNX2-luc activity data for these three compounds. No other compounds added to OM led to increased calcium deposition by BMMSCs over OM-only conditions (Figures 3D–I). Interestingly, addition of either curcumin (Figure 3F) or sulforaphane (Figure 3I) actually resulted in significant suppression of calcium deposition in a dose-dependent fashion, which correlates with the hRUNX2-luc activity for these two compounds (Figures 1F,I). These results demonstrate that apigenin and baicalein exert potent *in vitro* functional osteogenic properties extending to mineralization similar to daidzein, a known osteogenic phytoestrogen.

### hRUNX2-luc Reporter Activity but Not ALP Activity Is Highly Correlated With *in vitro* Mineralization Assay

**FIGURE 4:**
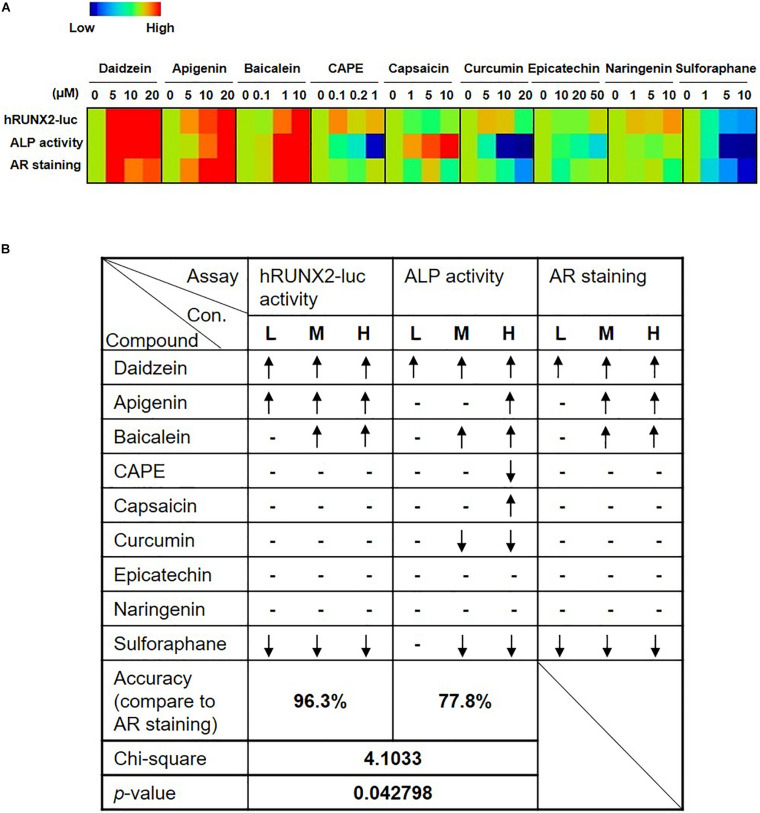
Comparisons of hRUNX2-luc reporter activity in C3H MSCs to *in vitro* functional assays demonstrate that hRUNX2-luc reporter activity but not ALP activity is highly correlated with *in vitro* mineralization assay. **(A)** Heatmap analysis of expression levels of hRUNX2-luc reporter activity in compound-treated C3H MSCs, and ALP activity as well as AR staining in primary murine BMMSCs treated with tested compounds. **(B)** Correlation analyses of expression trends in all three *in vitro* assays to mineralization assay for each tested compound. When compared to OM-cultured MSCs without compounds, significant increases in specific assay endpoints in compound-treated conditions were labeled as “↑,” while significant decreases were labeled as “↓,” and no changes (non-significant increases or decreases) are labeled as “–.” *p* < 0.05 using goodness of fit.

To assess the robustness of the rapid *in vitro* hRUNX2 transcriptional activity screening platform, we compared the outcome in all three *in vitro* osteogenesis assays for all nine tested compounds: the hRUNX2-luc promoter assay conducted in C3H MSCs, ALP activity conducted with primary BMMSCs, and mineralization conducted with primary BMMSCs. Using a heatmap graph to visualize these comparisons, we found that the best correlation was between the hRUNX2 transcriptional activity and mineralization assay, with nearly all tested compounds demonstrating similar trends between the two assays except for the compound naringenin (Figure 4A). Surprisingly, ALP activity correlated poorly with the other two assays: only four out of nine compounds—daidzein, apigenin, baicalein, and sulforaphane—yielded ALP activity results that were similar to hRUNX2-luc activity and/or calcium deposition assay. ALP activities of four other compounds—CAPE, capsaicin, curcumin, epicatechin—trended in the opposite direction of the other two assays; only with one compound, naringenin, was there some correlation of ALP activity to hRUNX2 transcriptional activity. To examine the predictive ability of hRUNX2-luc transcriptional activity for osteogenesis, we further calculated the correlation based on the trends of compound effects on MSC osteogenesis with the results in mineralization (quantified AR staining), which is the most finite functional assay for *in vitro* bone development, and found that results in hRUNX2-luc reporter assay had a significantly positive correlation with AR staining at 96.3%, which was better than the correlation of ALP activity—a functional, early marker of osteogenesis—with AR staining which was only 77.8% (Figure 4B). These results demonstrate that the rapid *in vitro* hRUNX2-luc activity screening platform is highly correlated with the more definitive and late-stage functional assay of mineralization, while the early osteogenic event of ALP activity poorly correlated with either the hRUNX2 transcriptional activity or mineralization assay.

### Compounds Screened Through the Murine C3H-hRUNX-Luc System Strongly Induce *in vitro* and *in vivo* Osteogenesis in Human BMMSCs

**FIGURE 5:**
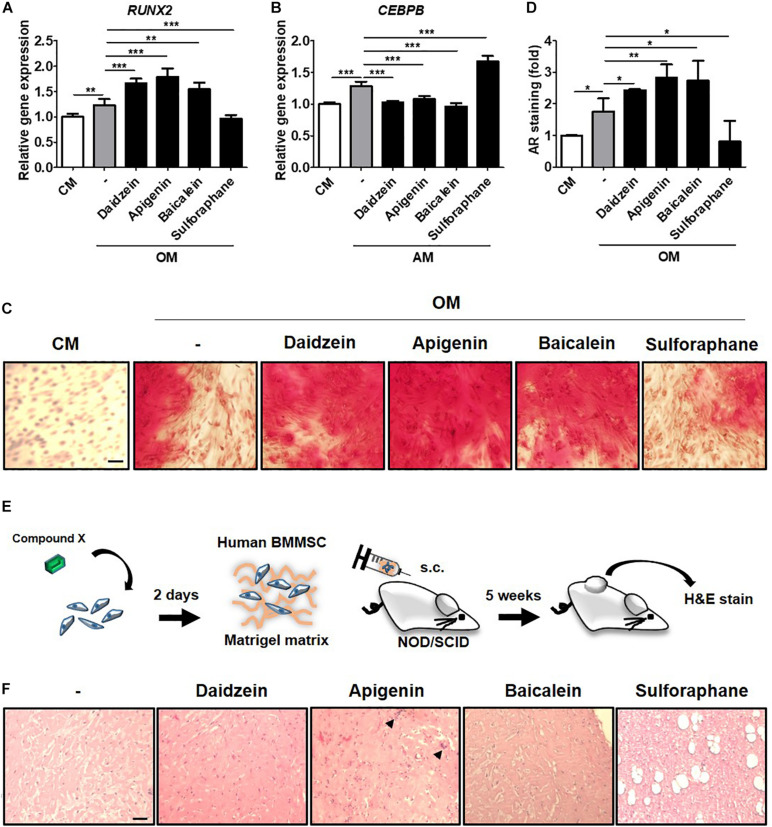
Compounds screened through the murine C3H-hRUNX-luc system strongly induce *in vitro* and *in vivo* osteogenesis in human BMMSCs. **(A)** Quantitative PCR (qPCR) analyses of gene expression of osteogenic master gene, RUNX2, was assessed in human BMMSCs under CM or OM with or without daidzein (10 μM), apigenin (20 μM), baicalein (10 μM), or sulforaphane (10 μM). Gene expression of each condition was normalized to the gene level in CM-culturing human BMMSCs (*n* = 5 for each group). **(B)** qPCR analysis of early adipogenic gene, CCAAT/enhancer-binding protein beta (CEBPB), was assessed in human BMMSCs under CM or adipogenic medium (AM) with or without daidzein (10 μM), apigenin (20 μM), baicalein (10 μM), or sulforaphane (10 μM). Gene expression of each condition was normalized to the gene level in CM-culturing human BMMSCs (*n* = 5 for each group). **(C)** and **(D)** Representative and pooled quantitative data (*n* = 4 for each group), respectively, of calcium deposition in human BMMSCs were assessed by staining for AR. Human BMMSCs were cultured in CM or OM with daidzein (10 μM), apigenin (20 μM), baicalein (10 μM), or sulforaphane (10 μM) for approximately 3 weeks, and AR staining was performed to assess extracellular mineralization by image examination **(C)** as well as AR elution and then quantification with normalized to the AR amount in CM-culturing human BMMSCs **(D)**. Scale bar, 50 μm. Data are expressed as mean ± SD. *, *p* < 0.05; **, *p* < 0.01; ***, *p* < 0.001. **(E)** Schematic diagram illustrating the *in vivo* ectopic bone formation: human BMMSCs were treated with selected compounds for 2 days and then mixed with Matrigel matrix for subcutaneous transplantation in immunodeficient NOD/SCID mice with H&E analyses of the implants at 5 weeks. **(F)** Assessment of osteoid formation by H&E staining; scale bar, 100 μm. Arrows denote multinucleated osteoclast-like cells.

To validate whether results from the C3H-transduced hRUNX2-luc reporter system and primary murine BMMSCs were relevant in the human system, we assessed the screened compounds for the ability to induce RUNX2 gene expression and mineralization in primary isolated human BMMSCs. We selected the most potent osteogenic compounds screened through the C3H-hRUNX2-luc assay and validated in the murine BMMSC mineralization assay—apigenin and baicalein—along with daidzein as the positive control, and also included sulforaphane as a negative osteogenic compound based on the murine MSC data. We found that addition of apigenin or baicalein, as well as daidzein, significantly increased RUNX2 gene expression levels in OM-cultured human BMMSCs at 48 h compared to OM-only conditions, while sulforaphane significantly decreased RUNX2 expression levels to below OM-only levels (Figure 5A). In contrast, addition of either of the three osteogenic compounds resulted in significantly decreased expression of C/EBPβ, one of the earliest transcription factors of adipogenesis (Guo et al., 2015), in AM-cultured human BMMSCs at 48 h to basal levels; interestingly, sulforaphane significantly increased C/EBPβ expression levels to levels above AM-only conditions (Figure 5B). *In vitro* mineralization assay using human BMMSCs demonstrated that addition of daidzein, apigenin, or baicalein significantly enhanced calcium deposition in OM-cultured human BMMSCs compared to OM-only conditions, while sulforaphane significantly suppressed mineralization to below OM-only levels and nearly down to undifferentiated conditions (Figure 5C, representative data; and Figure 5D, pooled quantitative data).

**FIGURE 6:**
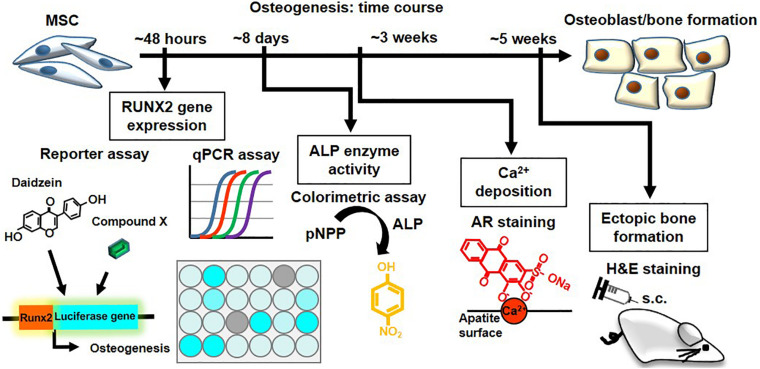
The hRUNX2-luc MSC-based *in vitro* platform is a rapid and robust system for screening of osteogenic compounds. Summary and time required of various osteogenic assays performed in the study: rapid screening for osteogenic phytoestrogens and natural plant-derived compounds using C3H-RUNX2-luc platform at 48 h with qPCR confirmation for RUNX2 gene expression. Validation using classical functional assays of osteogenesis using primary murine and human BMMSCs was performed for the early-stage assay of *in vitro* ALP activity which required 8 days, late-stage assay for *in vitro* mineralization assay which required 3 weeks, and *in vivo* ectopic bone formation which required 5 weeks.

To assess the *in vivo* relevance of these collective *in vitro* findings, we assessed for induction of ectopic bone formation using human BMMSCs in immunocompromised mice (Figure 5E). We found that subcutaneously transplantation of human BMMSCs pretreated with daidzein, apigenin, or baicalein improved osteoid formation compared to vehicle-treated BMMSCs, whereas pretreatment with sulforaphane enhanced adipogenesis rather than osteogenesis (Figure 5F). Interestingly, apigenin pretreatment seem to induce more mature bone/osteoid formation, with multinucleated osteoclast-like cells seen in sections. These results show that apigenin and baicalein are potent plant-derived osteogenic compounds for human BMMSCs, whereas sulforaphane inhibits osteogenesis and may be an inducer of BMMSC adipogenesis. Collectively, these findings demonstrate that the hRUNX2-luc cell-based *in vitro* platform is a highly predictive and robust system for rapid screening of osteogenic compounds (Figure 6).

## Discussion

The increasing incidence of OP globally have been accelerating, and while a number of treatments are available, compliance continues to be a major problem due to slow efficacy and rare but serious side effects (Warriner and Curtis, 2009; Hiligsmann et al., 2019). Human dietary studies have demonstrated phytoestrogens and other natural plant-derived compounds to have minimal safety concerns, but while the chemical structure of individual compounds is helpful for inference of estrogenic effects, dose-related differences in potency and efficacy has been difficult to predict, hampering drug discovery and development (Dang and Lowik, 2005; Vitale et al., 2013). Such challenges are further compounded in screening for osteogenic effects by the long process required for both *in vitro* and *in vivo* functional assays—which range from several days for the ALP assay to several weeks and even months for mineralization assays—with the additional requirement of using primary cells with the capacity for osteogenesis. Indeed, one major reason for the conflicting reports of agonistic/antagonistic osteogenic properties of many compounds may be due to the quality of the primary MSCs used, since it is well documented that MSC senescence is related to a loss of osteogenic differentiation capacity while increasing adipogenic capacity (Nuttall and Gimble, 2000; Moerman et al., 2004; Ho et al., 2013; Conley et al., 2020). We therefore sought to develop a rapid and robust *in vitro* platform for drug screening of osteogenic compounds based on our previous work on the proximal human Runx2 promoter, the master osteogenesis transcription factor, introduced into an immortalized, non-cancerous MSC cell line. Validation performed with primary isolated BMMSCs, the progenitors of osteoblasts, of classical *in vitro* functional assays including ALP activity and mineralization as well as *in vivo* osteogenesis demonstrated high fidelity of the hRUNX2-luc assay to accurately select osteogenic compounds with dose-dependent information at a fraction of the time and effort required for osteogenic functional assays.

Given the high incidence of OP worldwide and severe adverse complications, there has been surprisingly only two reports on developing *in vitro* platforms to screen for osteogenic compounds (Hojo et al., 2008; Li et al., 2009). Strangely, these two previous reports used rodent promoters of the osteogenic genes collagen one and BMP2 as screening platforms, rather than the corresponding human promoters. The choice of collagen one as a screen for osteogenesis is likely too non-specific, since this protein is the most abundant proteins in humans (Di Lullo et al., 2002). Conversely, while BMP2 is clearly a strong osteogenic agonist and has been therapeutically available as a recombinant protein for many years, there has been a number of clinical reports on the considerable side effects associated with this molecule (Carragee et al., 2011). Moreover, major pathways involved in osteogenesis including BMPs—as well as estrogens/sex hormones, Wnt/β-catenin, and sirtuins—are clearly also important in other biological processes (Varga and Wrana, 2005; Bartoli-Leonard et al., 2018); thus, screening platforms based on these pathways can lead to non-osteogenic effects. Since all of these major osteogenic pathways have been found to converge on RUNX2 for osteogenic effects, the use of transcriptional activity of this osteogenic master transcription factor as a screen for drug discovery would appear to be highly efficacious. An additional benefit of using Runx2 activity as an outcome may be the ability to predict adipogenic differentiation—as was seen with the compound sulforaphane (Figures 1I, 2I, 3I, and 5F)—which has confounded the therapeutic value of a number of phytoestrogens for osteogenesis (Dang and Lowik, 2005; Vitale et al., 2013). Our use of the Runx2 promoter as a screening tool, therefore, has the strong advantage of capturing compounds acting as agonists in any of the major osteogenic pathways as well as excluding compounds that induce adipogenesis.

Data obtained from this screening platform likely have strong translational implications since we have diligently performed several *in vitro* functional assays using murine as well as human primary isolated BMMSCs, along with an *in vivo* assay using human BMMSCs, which previous reports have surprisingly not done, bringing to question the robustness of those screening platforms. Interestingly, we found that the ALP assay was not as consistent or robust as the *in vitro* mineralization assay or the *in vivo* osteogenesis assay, the latter two assays which arguably offer more definitive information by evaluating osteogenesis to the outcome of mineralization. The wide distribution of ALP in many organs/tissues is well known and may be reason for the less specific correlation of this assay to mineralization assays. Given that the data from the hRUNX2-luc platform correlate best with the mineralization assays rather than the ALP assay, it appears that the hRUNX2-luc screening platform is robust at selecting osteogenic compounds.

In summary, we developed a rapid *in vitro* platform for screening of phytoestrogens and other natural plant-derived compounds using the hRUNX2-luc reporter transduced into an MSC line. Multiple functional assays both *in vitro* and *in vivo* and using primary isolated BMMSCs from both murine and human systems were performed for validation, with results demonstrating robust correlation from this *in vitro* screening platform. These findings implicate the contributions of a rapid and highly predictive *in vitro* screening platform using MSCs toward discovery of potent therapeutic candidates in the global fight against OP.

## Data Availability Statement

The original contributions presented in the study are included in the article/Supplementary Materials, further inquiries can be directed to the corresponding author/s.

## Ethics Statement

The animal study was reviewed and approved by the Laboratory Animal Center, National Health Research Institutes.

## Author Contributions

M-LY: conceptualization and funding acquisition. L-TW, Y-WL, C-HB, H-CC, H-HW, and BY: methodology. L-TW, Y-WL, H-CC, and H-HW: investigation. L-TW, BY, and M-LY: writing—original draft. L-TW and M-LY: writing—review and editing. All authors contributed to the article and approved the submitted version.

## Conflict of Interest

The authors declare that the research was conducted in the absence of any commercial or financial relationships that could be construed as a potential conflict of interest.

## Abbreviations

OP: osteoporosis
HRT: hormone replacement therapy
SERMs: selective estrogen receptor modulators
MSCs: mesenchymal stem cells
BMPs: bone morphogenic proteins
hRUNX2-luc: human RUNX2 promoter
C3H: C3H10T1/2
BMEM: Basal Medium Eagle medium
BM: bone marrow
CM: complete medium
DMEM: Dulbecco’s Modified Eagle’s medium
DMSO: dimethyl sulfoxide
CAPE: caffeic acid phenylethyl ester
MTT: 3-(4,5-dimethylthiazol-2-yl)-2,5-diphenyltetrazolium bromide
pCMV- β-gal: CMV-driven β-galactosidase construct plasmid
OM: osteogenic medium
AM: adipogenic medium
pNPP: p-nitrophenylphosphate
ALP: Alkaline phosphatase
AR: Alizarin Red
H&E: hematoxylin and eosin.
