# Supplementary material for: A Rapid and Highly Predictive in vitro Screening Platform for Osteogenic Natural Compounds Using Human Runx2 Transcriptional Activity in Mesenchymal Stem Cells
Source: Front Cell Dev Biol. 2021 Jan 8;8:607383. doi: 10.3389/fcell.2020.607383 (PMC7849832; doi:10.3389/fcell.2020.607383)
Supplement: Supplementary file 1 [file Data_Sheet_1.PDF]

Supplement

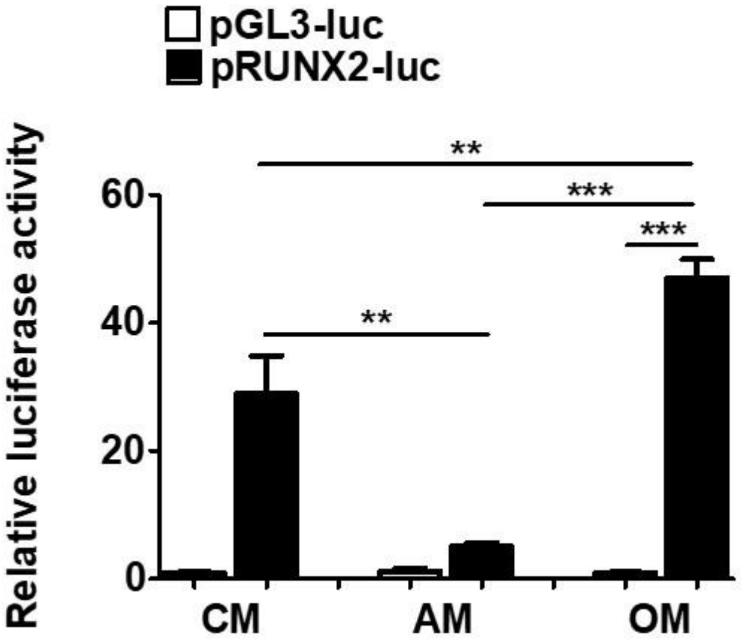

Figure S1. Human RUNX2 (hRUNX2) promoter activity in murine C3H10T1/2 (C3H) mesenchymal stem cell (MSC) line under control/expansion conditions, osteogenic, and adipogenic differentiation conditions. Establishment of a cell-based high-throughput method for screening osteo-responsive compounds. The murine C3H MSC line was transfected with the hRUNX2 promoter-luciferase reporter plasmid (hRUNX2-luc) or vector only (pGL3-luc), along with CMV-driven β-galactosidase construct plasmid (n = 3 for each group). After 24 hours, the culture medium was changed into CM, AM or OM and cells were assessed for luciferase activity 48 hours later (please see Materials and Methods for experimental details). Data are expressed as mean ± SD. **, $p < 0.01$; ***, $p < 0.001$.

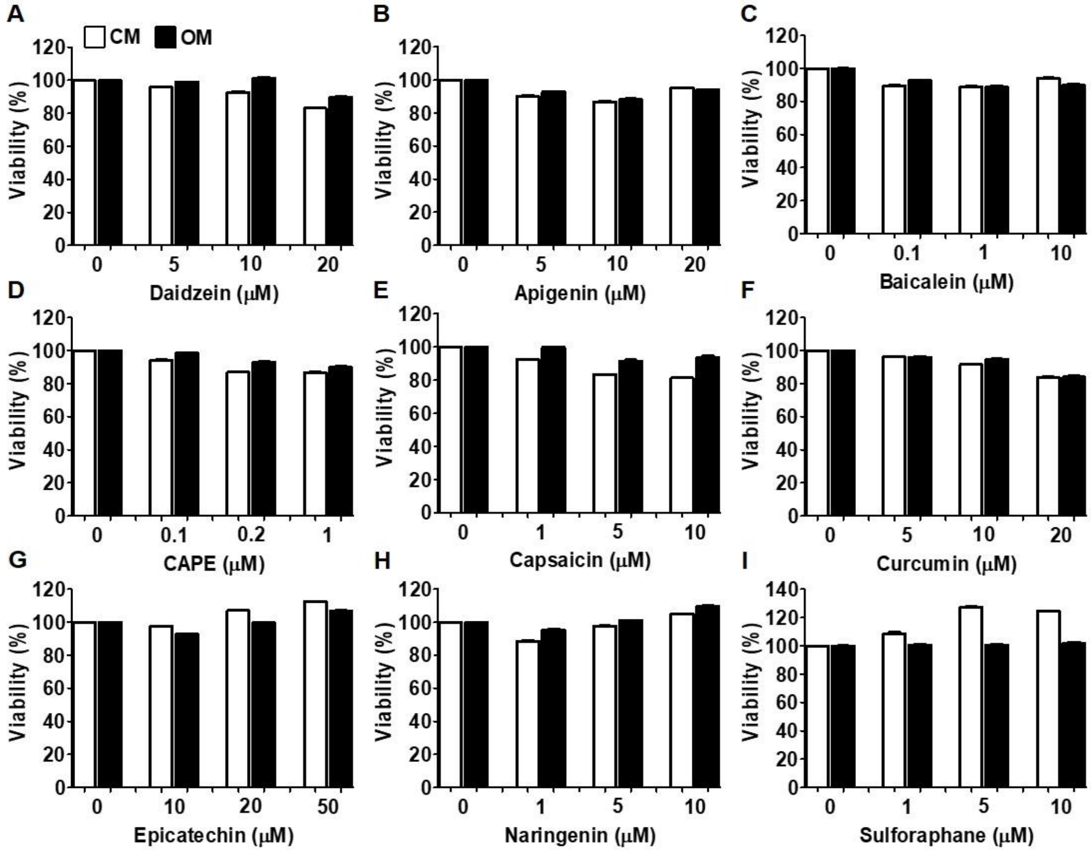

Figure S2. Cytotoxicity assessment of tested compounds on MSC viability. Cytotoxicity of the following compounds was assessed in CM- or OM-cultured C3H MSCs by MTT assay: (A) 5, 10 and 20 μM of daidzein, (B) 5, 10 and 20 μM of apigenin, (C) 0.1, 1 and 10 μM of baicalein, (D) 0.1, 0.2 and 1 μM of CAPE, (E) 1, 5 and 10 μM of capsaicin, (F) 5, 10 and 20 μM of curcumin, (G) 10, 20 and 50 μM of epicatechin, (H) 1, 5 and 10 μM of naringenin, or (I) 1, 5 and 10 μM of sulforaphane (n = 3 for each group).
